# Exploring the Caregiver Role after Deep Brain Stimulation Surgery for Parkinson's Disease: A Qualitative Analysis

**DOI:** 10.1155/2023/5932865

**Published:** 2023-04-05

**Authors:** Suzette Shahmoon, Patricia Limousin, Marjan Jahanshahi

**Affiliations:** Unit of Functional Neurosurgery, Department of Clinical and Movement Neurosciences, UCL Queen Square Institute of Neurology, 33 Queen Square, London WC1N 3BG, UK

## Abstract

This pilot study aimed to explore how caregiver spouses make sense of themselves one and five years after their partner's deep brain stimulation (DBS) surgery for Parkinson's disease. 16 spouse (8 husbands and 8 wives) caregivers were recruited for the interview. Eight struggled to reflect on their own lived experience and primarily focused on the impact of PD on their partners, such that their transcripts were no longer viable for interpretative phenomenological analysis (IPA). A content analysis showed (1) how these 8 caregivers shared less than half as many self-reflections than the other caregivers, (2) that there was a bias to reflect on their partner's experience answering the opening question, (3) the bias continued when answering subsequent questions, and (4) there was a lack of awareness of this bias. No other patterns of behaviour or themes were able to be extracted. The remaining 8 interviews were transcribed and analysed using IPA. This analysis discovered 3 inter-related themes: (1) DBS allows carers to question and shift the caregiver role, (2) Parkinson's unites and DBS divides, and (3) seeing myself and my needs, DBS enhances visibility. How these caregivers interacted with these themes depended on when their partners were operated. The results suggested that spouses maintained the role of caregiver one year post DBS because they struggle to identify themselves in any other way but were more comfortable reassociating into the role of spouse 5 years post surgery. Further inquiry into caregiver and patient identity roles post DBS is recommended as a means of supporting their psychosocial adjustment after surgery.

## 1. Introduction

Deep brain stimulation (DBS) is effective in controlling the motor symptoms of Parkinson's disease (PD) for 5 or more years [1, 2]. DBS has been described as creating a “biographical disruption” for the patient as it can change the way they interact in the world quite suddenly once PD symptoms are improved [3]. This biographical disruption can be associated with poor psychosocial adjustment post DBS [4] due to the sense of loss related to the care once received in comparison to the independence possible post DBS [5] and challenges around identity regarding the merging with technology [3, 6, 7].

The DBS journey is thought to be comprised of 4 stages [8]. The presurgery stage focuses on the decision-making process patients and their caregivers use to decide to opt for DBS surgery. The second stage focuses on the surgery itself and the physical support needed along with the clinical goal-setting. The third stage focuses on the changes that DBS creates in symptoms, behaviours, and roles. The fourth stage refers to the patient's reengagement with life as well as their perceptions of the future. These third and fourth stages are the most relevant with regards to how life is experienced 1 and 5 years post DBS. The impact of DBS surgery has yet to be explored with regards to shifts in caregiver role and identity over time after the surgery.

According to the “social identity theory,” we make sense of ourselves in the context of the social groups we ascribe to, and how we engage in the behaviours we consider appropriate to those groups [9]. Over time, spouses, partners, and other family members who care for an ill relative, become depended upon for care, practical, and emotional support, potentially emphasizing the social role of the caregiver [10, 11]. As PD progresses, it can become harder for caregivers to remain employed, socialise independently, and feel connected to others [12, 13]. This can reinforce the caregiver role as it becomes more challenging to engage in any other social role(s) [14].

The caregiver identity theory suggests that there are 5 phases which caregivers can transition through during their caregiving “career” [15]. The first two phases are comprised of the caregivers becoming more involved in care which extends beyond a family, or a friendship role, which starts the process of identifying with the role of caregiver. In phase three, carers tend to be more involved in such activities as personal grooming of the ill person. This can put both parties ill at ease, and the individual is most likely now to identify with the role of caregiver around fifty percent of the time. Caregivers enter phase four when their lives are heavily dominated by their caregiving role. Phase five is characterised by the reverting back to an earlier phase of caregiver identity, thanks to a change in circumstances. DBS may be one such circumstance.

Caring for a spouse can be physically, emotionally, socially, existentially, and financially demanding and can leave caregivers feeling isolated, invisible, and in-need [16]. The concept of caregiver burden is not new and has been evaluated in many studies of PD [17]. There are also countless studies that have examined the effect of caregiving on identity [18]. Many studies have focused on the identity change perceived by caregivers of their caree [19]. Other studies have looked at how these changes can be managed and facilitated (Haahr et al. [20]). Most studies tend to focus heavily on the caregiver's experience of the person with PD with whom they live and the way that has impacted their own daily life. Themes have emerged such as “A sense of freedom embracing life” (Haahr et al. [21]). This theme focuses on the shifts in the patient's physical state which have given caregivers more freedom. Restoration of the “old self” [22] gives insight into the return of the person with PD's functional abilities and mobility and how that affects their personality. Themes such as, “being different after DBS” [23], “clinical management of personality change” [22], and “the challenge of changes and constraint” (Haahr et al. [21]) all focus on the adjustments needed to be made by caregivers to manage the changes DBS has made to the person with PD. All of these studies have given us important insights into how caregivers experience the changes in their caree's physical, psychological, and emotional state. However, there has been little exploration of how caregivers perceive their own evolution as individuals and their caregiver role. Caregivers can feel a sense of loyalty towards the person they care [24], which can overshadow their own needs [16]. By maintaining so much focus on their caree, there is a lack of information regarding the caregiver's personal journey. This pilot study aimed to fill this gap in the literature by investigating how caregiver spouses make sense of themselves, as well as their caregiving role one and five years post DBS surgery.

## 2. Methods

### 2.1. Design

This study used semistructured interviews employing an interpretative phenomenological analysis (IPA) (Smith et al. [25]) approach to understand the lived experience of caregivers 1 year or 5 years after their spouse's DBS surgery to treat PD. IPA was the analysis of choice due to the double hermeneutic that is used in its approach. With each question posed, participants are invited to reflect and make sense of their lived experience. They are given the space to reflect about how they think and respond to the circumstances of their life. As they share these reflections, the researcher is then invited to do the same (Smith and Shinebourne [26]). A summative content analysis [27] was used for those interviews which did not meet the threshold of caregiver personal reflections needed for IPA, as it displayed the measurable differences between those caregivers who shared self-reflections and those who were more restrained.

### 2.2. Participants

IPA requires a relatively small and homogenous sample. An equal number of male and female spouses were recruited. Participants were heterosexual, British, or Irish, over the age of 50, married for at least 10 years, and living with their spouses who were diagnosed with PD at least 10 years prior to interview. Spouses with PD had bilateral DBS of the subthalamic nucleus (STN) and were either 1 or 5 years post DBS at the time of interview. All caregivers who met the recruitment criteria were contacted from the patient list of the Functional Neurosurgery Unit at the National Hospital of Neurology and Neurosurgery in London. However, to ensure a sample size of 16, the threshold of time post surgery was changed to 12–18 months and 4-5 years.

Caregivers who met the recruitment criteria were contacted by phone or e-mail and were invited for interview. 16 participants were interviewed in total; 8 participants were interviewed 12–18 months post their spouse's surgery, and 8 participants were 4-5 years post their spouse's surgery.

### 2.3. Rapport Building

In a study employing interview methodology, an essential element of data collection is rapport building. It has been noted that people who share similar experiences of illness can create a unique connection because they are able to give instrumental advice and emotional support as their empathy comes from a place of shared knowledge [28]. This has been researched in the realms of peer support, but the same principles can be true for qualitative research, and hence can help facilitate interviews.

For this reason, the interviewer disclosed to each participant that she had experience of caring for a spouse with PD who had undergone DBS surgery. This information was welcomed by the participants, and many reflected that it was comforting to speak to someone with similar lived experience. While this disclosure by the interviewer seemed to lift some barriers with regards to self-disclosure by the caregivers, it may have created other challenges as some participants seemed to expect the interviewer to understand their experiences without them having to share details. This meant that in such cases, the interviewer had to make a greater effort to elicit the detailed responses required for IPA.

### 2.4. Procedure

Ethics approval was granted by the HRA and Health and Care Research Wales (REC Ref 18/LO/1368). Informed consent was obtained from all participants.

A semistructured interview schedule of 10 questions with prompts was devised, aimed at understanding the lived experience of caregiver spouses approximately 1 and 5 years post their spouse's DBS surgery. A pilot interview was held, and the interview schedule adjusted to make sure that the questions were clear enough for the participants to understand. A set of prompts were used to help guide the participants reflect on the questions.

An example of this is with the question, “How does DBS affect how you think about your partner who has PD?” The following prompts were used: “Has the surgery affected the way you view your partner? Has DBS affected how you care and your involvement in their care? How has that affected the way you feel about them? Has that been a difficult change?”

Interviews lasted around 60 minutes. Interviews were conducted by SS. 3 interviews took place in the participant's homes. The participants arranged to be alone at home to allow for maximum comfort with regards to disclosure. The remaining 13 interviews took place in the privacy of the hospital clinic. All interviews were recorded and transcribed verbatim. All names were changed to codes of which the first was the letter W or H depending on if they were a husband or wife, a number, and then Y1 or Y5 depending on when their spouse was operated. Any identifying information was removed to protect confidentiality.

### 2.5. Data Analysis

#### 2.5.1. Interpretive Phenomenological Analysis

The first stage of analysis was conducted following the guidelines set out by Smith, Flowers, and Larkin (Smith et al. [25]). Each interview was analysed in isolation of the others to maintain objectivity regarding each participant's lived experience. Interviews were subjected to an inductive process which started by annotating transcripts with initial observations in the margin. These notes were then converted into “personal experiential statements,” (PES's) in the other margin of the transcript.

Furthermore, analysis made it clear that the caregivers could be distinguished into two groups. The interviews of the first group had in excess of 50 PES's (group 1, *n* = 8) and were analysed using IPA. Those with less than 30 PES's (group 2, *n* = 8) did not provide enough data for an IPA analysis. Changing the style of analysis to fit the data was not an option as this lack of self-reflection shown by group 2 was worthy of note, and hence their interviews were analysed using a content analysis [29].

All of the PES's for group 1 were collated, and connections between the various statements were considered and clustered into groups. “Personal experiential theme's” (PET's) emerged for each cluster. All of the PET's were then collated and clustered according to the connections that could be made between them, and 2-3 superordinate themes per transcript emerged. At each stage of analysis, checks were conducted by other members of the research team to make sure the first author was not projecting any of her own personal experiences on the data.

A table for each transcript's superordinate themes, with the relevant PET's, PES's, and supporting statements from interviews was created.  Table 1 presents an abbreviated example taken from H3Y1's interview. The first superordinate theme that emerged from the interview was, “If she's well, I'm well.” This theme emerged from the two PET's, “Living a combined life” and “The caring caree irrespective of DBS.” 2 supporting PES's and keywords have been shown to exemplify the process.

These tables were used to compare across cases. All of the superordinate themes from the year 1 interviews were clustered together from which 3 themes emerged. The first focused on identity, the second on individuality, and the third on personal needs. When the year 5 superordinate themes were clustered, a similar pattern emerged allowing for the 3 main themes presented in Table 2, to emerge.

Tables were created, for each of these themes, with the supporting superordinate theme, PET and PES from each individual interview in one column, and supporting quotes in the adjacent column. These tables were split into two sections: 12–18 months post DBS (Group A) and 5 years post DBS (Group B).

#### 2.5.2. Content Analysis

Considering that the focus of this study is on identity, the content analysis took place in two stages. The first stage of analysis was comprised of quantifying how much these caregivers shared their own life experience by counting PES's compared to the caregivers in group 1.

The second stage of the content analysis was aimed at looking for any other patterns in the data which could help us to make sense of these caregiver's experiences. Unfortunately, very few meaningful patterns emerged beyond the ways in which these participants answered the questions posed, due to a lack of data.

## 3. Results

Data analysis revealed that two groups of caregivers could be distinguished as follows:

  Group 1: those caregivers who had above 50 personal experiential statements during the interview
  Group 2: those caregivers who had below 30 personal experiential statements during the interview

### 3.1. Group 1: Caregivers Who Expressed Their Own Personal Experiences of Caregiving

Table 3 shows the three themes that emerged across post-DBS year 1 and year 5 interviews.

The focus of this paper is on how spousal caregivers redefine their self within their caregiver role post DBS. PD shifts the way caregiver spouses see themselves, their partners, and their roles within their marriages. DBS causes new shifts, forcing them once more to review all these aspects.

It has often been reported that people with chronic illness adopt a sick role and caregivers adopt a caregiver role. However, DBS has the potential to improve the physical symptoms of PD almost overnight which can alter how these two roles proceed to manifest following surgery. These interviews highlight elements of the journey the spouses interviewed had taken in their caregiving role post DBS surgery. Each of the themes that emerged will be presented in two sections: 12–18 months and 5 years post-DBS. Each group was comprised of 2 wives and 2 husbands. Comparisons between these groups will be focused on in the discussion.

#### 3.1.1. 12–18 Months Post DBS

Theme 1: DBS allows carers to question and shift the caregiver role.

Am I more than just a caregiver?

All 4 of the spouses who were interviewed 12–18 months post DBS stopped working prior to surgery so that they could care for their partners, reinforcing the caregiver role. W2Y1 describes a newfound sense of freedom since her husband's surgery because,

*“It gave me a bit more freedom, probably, so I didn't feel so bad about going out and doing things and I joined the gym and yeah, I joined the gym, and I, that's when I went back to teaching”*

We note here the guilt W2Y1 alludes to having experienced prior to surgery, when she went out without her husband. It is not clear if there is an effect of gender, but in this study, there was a difference in how the carer husbands reacted to their caregiving carees compared to the wives interviewed.

*“I have worked since I was 16, in fact before that really, so suddenly I didn't have that role and then there was a possibility that I didn't have another role so that was a bit tricky, it soon became apparent that I at least had one of those roles.”* H1Y1

H1Y1 describes how he completely surrendered to his caregiving role. Everything he did was for his wife, freeing him from any guilt. For as much as it was a smooth transition from his working role to his caring role, it is the potential disruption to his caregiving role that he struggles with. We see from the previous quote, H1Y1 is so entrenched in his caregiver role, he struggles to imagine life without it now that he no longer works. The potential success of DBS would have left him feeling redundant and in search of a new way of seeing himself.

W4Y1 showed more resentment towards her situation than H1Y1 when she states:

*“I don't think he does understand, he'll say to me I've got Parkinson's, yes he has but he, I don't think he realizes that actually it's not just him that's got Parkinson's and I could, I could have not stopped working, I could have carried on working.”*

W4Y1 is not only angry with the effects PD is having on her life, she is struggling because her husband seems “blind” to her suffering and the loss she has experienced by giving up work. When she says, “*it's not just him that's got Parkinson's.”* She is identifying with her husband's illness because PD is affecting her life as much as it has affected his.

Theme 2: Parkinson's unites and DBS helps divide.

The combined self as follows:

W4Y1 has just shown us how PD seems to create a sense of merged self, whereby the caregiver starts identifying with their spouse's illness. The caregiver's life can be so governed by their partner's condition that all four caregivers identified with their partner's illness and sick role as well as their caregiver role. This identification with the PD role is well displayed in both husbands' narratives.

H3Y1 describes his lived state as a “combined self,” suggesting that he too fails to see himself as an autonomous individual. He states, *“We actually say, you know, we've got Parkinson's, if you like, so it's more inclusive.”*

Unlike W4Y1 who is identifying with the sick role because of her own inner pain, H3Y1 is identifying with his wife's PD out of solidarity. He wants us to know that he is not just supporting his wife on her journey, he is very much a part of her PD journey. PD tends to make the patient feel isolated, H3Y1 does not want his wife to feel alone in her suffering hence he states, “we have Parkinson's.”

H1Y1 shares how he experiences this phenomenon, when describing his fears around DBS as follows:

*“I do tend to see myself through (my wife's) lens really in the sense that I'm kind of basically about her, really, and I have been for a long time. I mean this is one of the things that people go, “oh you're a carer” and I am a carer and I do feel like I'm a carer.”*

To an extent, this explains H1Y1's relief that DBS did not give his wife full independence, since his caregiver role forms a major component of his current identity. He may not be as explicit as to state that he has PD, but caring for his wife seems to give him a sense of purpose. “I'm kind of basically about her” suggests that caring for her doesn't just make his life meaningful, it encompasses the full experience of his life, hence his reticence to relinquish this identity.

Caregiver spouses described how their lives were filled with many restrictions prior to DBS. They all described a reality where they were overcome by tending to the needs of their spouses, and hence their own needs became less visible. They were only able to focus on what PD allowed. DBS starts to shift this reality, in the first year, as it lessened the needs and demands of the PD patients on most of the spouses. We will see how this becomes more pronounced after 5 years.

Theme 3: Seeing myself and my needs, DBS enhances visibility.

I am still invisible, DBS has not helped enough.

When H1Y1 stated, *“I do tend to see myself through (my wife's) lens really in the sense that I'm kind of basically about her,”* we note that irrespective of DBS, all of his attention remains on his wife's needs.

DBS grants the PD patient improvement of their motor symptoms, and hence some independence has the potential to create a break in that combined self and allow caregivers the opportunity to tend to themselves but that means caregiver spouses need to become more self-aware. Caregivers would need to acknowledge the separation that has occurred between them and their partners post surgery, allowing them to see themselves and behave as separate entities. One could expect the success of surgery to determine the extent of this separation, as the carers and their needs become more visible. However, this had not happened for any of the caregivers who were interviewed 1-year post surgery irrespective of the success of surgery. Whether it was seeing friends, having time alone or just recognising their own self, most of the spouses struggled to recognise or address their needs or individual state 1-year post DBS.

H3Y1's wife has had a good result with DBS. She is much more mobile and independent which should allow him the freedom to see himself as an autonomous partner, allowing him to recognise and tend more to his own needs and desires. When asked to reflect on the best part of DBS and how this had impacted on his life he responded:

“*the best bit really is, is seeing her life get better, that's, and I know that's impacted, that's obviously good for me too but what I mean is, that's been the greatest pleasure to see that she can now do more, and the windows are not shutting quite as much as they were, on us, you know I wouldn't push off for a holiday or something and do something like that for myself if you like.*”

H3Y1 is so used to his “combined life,” he still only ever reflects on his wife's experience. When he says “*that's obviously good for me too*,” it is evident that any improvements that DBS has made to his life seem secondary. Throughout the interview, he expresses how he has never felt the need to address his needs, he places hers above his, and even now he shirks away from any mention of him doing so when he states, “*I wouldn't push off for a holiday or something and do something like that for myself.”* H3Y1 still focuses his attention on his wife and relies on her state to determine how he feels and functions in life.

Like H3Y1, W2Y1's husband has also had a good result from DBS, but she is more able to reflect on how it has impacted on how she experiences life.

“*After the DBS we started on a different sort of level, right, and where I felt more, I felt more freedom. And I didn't feel that I had to be constantly checking on hi*m.”

We see here that unlike H3Y1, W2Y1 is aware of the improvement DBS has made to her husband and the impact it has made on her own life. She is no longer needed to constantly keep a watchful eye on him. On the contrary, she can set her sights on herself. However, she also stated:

*“I was tired, and I just couldn't be bothered doing things and he was tired anyway with Parkinson's and he couldn't be bothered. We started to make excuses not to go out with our friends and we've always had a really, really busy social life. And, we've loads of friends and friends that we've had since school, and I just started to think, “I just don't have the energy anymore.” And, that's sort of stayed with me I haven't really come back from that.”*

We see how different life was for W2Y1 prior to PD and DBS. She and her husband enjoyed socializing with friends until PD stopped them. The fact that they have maintained their childhood friends shows to what extent these connections are meaningful. However, even though DBS had given her the freedom to contemplate bringing socializing back into her life, she was struggling. For W2Y1, reengaging with the world was reliant on the energy she has not recovered and consequently she is unable to socialise. DBS has given her clarity on what is important, but it has not given her the strength to go after it.

All year 1 spouses failed to change the way in which they engaged with the world even though DBS changed their circumstances. The positive shifts that had occurred within their physical lived experience had not trickled down to their emotional life. Some still struggled to see themselves as anything other than care partners, while others did not have the strength to change their way of being.

#### 3.1.2. 5 Years Post DBS

Theme 1: DBS allows carers to question and shift the caregiver role.

The carer identity has shifted.

All of the spouses in Group 1 who were interviewed 5 years post DBS, shared this ability to let go of the sense of responsibility to care for all of their partner's needs, allowing them to act like carers less and act like spouses more again. H4Y5 sought some support through a counsellor who helped him to recognise, 

By allowing his wife to support him, he has brought balance back into their relationship. They no longer interact as caregiver and recipient, there is more of an equal exchange of care and support.

The 2 wives interviewed in this group did not reflect so much on how their husbands could now support them in the way H4Y5 did. They both discussed how they made more of an assertive effort to make this shift out of their caregiver roles by addressing the ways in which they interacted with their husbands.

W1Y5 remembers pre-DBS,

*“It just put a strain because I became more of a carer, more than an equal and I obviously changed.”*

The strain of managing her husband changed how she saw herself. Now, 5 years since she says,

*“I make a conscious effort not to help him sometimes and to sit back and let him get on with things rather than, you know, before his DBS I used to have to help him out of the chair and now if he's struggling a little bit I will just let him get on with it.”*

DBS has relieved that sense of responsibility she once felt. She now allows herself to allow him to be more independent and gives herself the freedom to sit back, while he tends to his own needs.

W4Y5 has also handed responsibility back to her husband; however, this has more of an emotional than a physical responsibility. She stated as follows:

“*sometimes I feel like his carer, don't get me wrong, sometimes I feel like his carer and I do tell him, “I feel like your carer today rather than you wife,” um and he sort of says, ok I'll do this and tell me what I need to do to make you feel like my wife. Cos sometimes it just feels like that sometimes that I'm doing everything constantly, reminding him to take his tablets, reminding him to do this, reminding and I say to him, hold on this week has been like a carer, you need to sort yourself out, and then we go back to him being the husband that I first met.”*

Through her verbal communication about being treated as a carer or a wife, W4Y5 is making her husband feel responsible for the way he makes her feel through his actions. She points out to him the behaviours which cause those role shifts within their relationship so that he can make the changes that help them to maintain their spousal roles. DBS has given W4Y5 the space to make sense of how PD impacts her relationship with her husband, and now she works hard to make sure she is not robbed of her spousal role again.

Theme 2: Parkinson's unites and DBS divides.

DBS brings balance in relationships through acceptance.

The 4 spouses, in this group, showed no signs of identifying with their spouses' sick role in the way the year 1 spouses did. They seemed more confident about their partners being more responsible for themselves. As we saw when W4Y5 stated,

*“I say to him, hold on this week has been like a carer, you need to sort yourself out, and then we go back to him being the husband that I first met.”*

These spouses had more confidence asserting their autonomy.

The husbands described how these shifts in autonomy occurred in terms of practicality. H4Y5's wife started working from home post DBS, but surgery meant she was now able to share more in the daily chores, allowing there to be more of a sense of equality within the home at a practical level.

“*DBS prolonged the time that she could do that, so that was a good thing but that's, that's been a bigger shift, you know, in the last few years, in terms of the kind of practicalities and so on, if she was still trying to do the job, you know, I'd be doing more still but because she's gone and got more flexibility now, about, you know she's working at home a lot and so on, so umm, you know, in terms of, you know, cooking.*”

The wives described more of an emotional journey towards finding their own autonomy. W1Y5 shared:

*“it was after the DBS because I think what happened was I suddenly realised I had been carrying a lot of baggage and that was enough that (my husband) was better, and I had to do something and reclaim my life a little bit.”*

DBS alleviated the emotional “baggage” that weighed her down. She stated:

“*I needed to reclaim my life a little bit, so I decided to take a year out and spend more time at home, do more of the things that I enjoyed because I think that I had lost myself in the caring, mothering, and working role”*

“Reclaiming her life” appears to be about redefining who she is by her actions. Prior to DBS, she needed to fulfil many roles imposed on her: carer, worker, and mother. However, DBS helped facilitate a shift in W1Y5's behaviour, allowing her more time to do what she liked, allowing her to see herself as more than just a carer or mother, and distancing her enough from the PD life and her husband's sick role, so that these no longer dominated her life.

Theme 3: seeing myself and my needs, DBS enhances visibility.

Acceptance means it is easier to live for me.

This shift in responsibility did not just allow spouses to redefine their sense of self, it also allowed them to start identifying and start addressing their needs, something which was evident in the previous section when W1Y5 started “reclaiming” her life.

H3Y5's wife did not feel a huge benefit from DBS. She still struggled with pain, mobility issues, and low mood. However, the small shift that did occur gave some relief to her husband, enough for him to start seeing his own needs.

Knowing she is more independent at home has given him the freedom to go to work and socialise with friends guilt free. He even expressed:

“*it got to the stage last year where I said look I need to get away I'm gonna go away for a week on my own*,”

Since his wife's DBS surgery, H3Y5 recognised his need for independence and his need for a holiday. DBS gave him the opportunity to address those needs.

H4Y5's wife was also struggling with mood and mobility issues. Unlike H3Y5, who has taken to socializing without his wife and going on holiday, H4Y5 had recognised a need for more emotional support.

*“as the effects of it have become more difficult in that period before DBS and again in the last couple of years I suppose that those, I think I've got better at talking to other people.”*

Prior to DBS, he described what it was like hiding his emotions thinking that was the best way of supporting his wife. However, DBS gave them a brief interlude, one in which he was able to become more self-aware and recognise his own struggles with the effects of the illness. As his wife's PD has started to progress, he now recognised his own needs for emotional support and has learnt to rely on his friends.

### 3.2. Group 2: Caregivers Who Did Not Express Their Own Personal Experiences of Caregiving

A content analysis was used to look for any other themes or patterns that could emerge from the interviews from the participants in group 2. The data collected was not rich enough to add anything to our understanding of caregiver identity post DBS surgery, even using a content analysis. Gender did not seem to have any effects on identity and, unlike group 1, neither did time since surgery. The location of where the interview took place did not seem to influence self-disclosure either, as one of the three participants who were interviewed at home was very comfortable sharing their experience and was hence in Group 1. The only overarching pattern worth noting was the strategies which these caregivers used in answering the questions posed, and Table 4 shows these three strategies. There was a constant desire by these participants to discuss their partners' lives rather than their own.

#### 3.2.1. Strategy 1: A Bias When Answering the Opening Question

The first interesting strategy of note was regarding how the participants answered the opening question. The interest and focus of the study on their own experiences as carers, was emphasized to the participants at the time of recruitment and before the interviews began. All the interviews were started by asking the participants,

*“Can you describe what life was like for you before your husband had DBS surgery, how did your spouse's Parkinson's disease impact on you?”*

H2Y5 answered,

*“The main problem was she had good and bad days, and the main problem was that a lot of the tablets wouldn't work because one of the symptoms of Parkinson's, she sweats a lot, so she'd have a very good day and a very bad day. She gave up work years ago.”*

W1Y1 answered,

*“It, I think it figured quite a lot in his decision to retire because he, as he says, knowing what he knows now, felt he had the symptoms 3 years before.”*

These quotations illustrate how both participants launched into descriptions of their partners' symptoms and reactions to those symptoms, when asked about their own experience as a caregiver. This initial response suggests that these caregiver spouses may have a natural bias to consider their spouse with PD before themselves. When their spouses had been operated, was of no consequence, none of the 8 of the spouses described the impact PD had on their lives, a phenomenon that did not occur in the other caregiver group.

When it was reflected back to each of these participants that they had shared their spouse's experiences, they were once again asked to consider and describe how PD had affected their own lives. Similar responses were given. As we see from the Table 5, only one wife fully engaged with the question and reflected on how her life had been changed by PD. One husband and one wife started to consider the effects of PD on their lives but then reverted to describing their spouses' symptoms. One wife deflected the question by describing the impact PD had on her children, and the other four participants in this group described once again, their partner's symptoms, with very little portrayal of how this had affected how they felt or operated in the world.

#### 3.2.2. Strategy 2: Continuation in Bias When Answering Subsequent Questions

As the interviews continued, this same pattern of behaviour persisted with all the participants. They either avoided, deflected, or reflected predominantly on their partners' lives. A good example of this is displayed by W1Y1.

“W1Y1: *he did say he thought he would be dead by last Christmas if he hadn't had the surgery and I absolutely believe he would have driven the car into a tree or a wall. I absolutely believe he would have done that.*

*Interviewer: How did that impact on you? Was there any impact on your relationship or you, knowing that you are out all day, you have got this stress, you have got this fear and then after a few years, once everything started to get worse, you have now got this added stress that he is telling you, I might not be here by next Christmas? How did that make you feel?*

*W1Y1: Well no he didn't, it was just the way he was, the not eating, he lost a lot of weight. I mean he could stand to lose some weight to be fair and he is a much better weight now than he was, so, but it seemed to be quite quick, it was quite sudden, now again with all the movement that there is with Parkinson's you are going to lose weight also the fact that he wasn't eating as he used to eat, which wasn't enormous plates full of food but a normal diet and that was worrying.”*

After describing her husband's symptoms, she shares the disturbing information that her husband had struggled with suicidal thoughts prior to DBS. When asked to reflect on how this impacted on her, her answer barely relates to the question asked. She seems to find solace in describing her husband's symptoms rather than spending too much time reflecting on her own personal thoughts and fears.

As the participants described their spouses' difficulties, a consistent effort was made by the interviewer to remind them that although their accounts were valid, they were being asked to consider how these difficulties impacted on their own emotional and physical wellbeing. 5 of the 8 participants spent minimal time self-disclosing and focused purely on their spouse as illustrated with W3Y1.

*“Interviewer: And how did that impact on your relationship with your husband? How was that for you?*

*W3Y1: Well I suppose he at times feels guilty that he has this need, and, but he has become, I suppose his personality is different than it would have been years ago, much more anxious about things, everything, and life is lived around Parkinson's really.”*

Two of the eight participants asked that one of their children be present during the interview. H4Y1's daughter interjected often during the interview when her father avoided questions. She made statements such as,

*“when it first happened, you were a bit, everyone was a bit sad at the start and then as it progressed sometimes it wasn't nice.”*

And

*“you do worry, don't you, you do worry because mum is, one thing you don't have to worry about drinking or like no health things.”*

Yet, even these prompts from his daughter did not inspire H4Y1 to share any details about his own experience.

#### 3.2.3. Strategy 3: Awareness of Bias When Answering Questions

Seven participants in this group seemed unaware of this bias towards describing their spouses' experience rather than their own. The one remaining participant in this group, outrightly stated that he preferred to speak about his spouse rather than himself. As we see from the following quote, his feelings seemed to be fully dependent on his wife's state of physical and emotional health.

*“Interviewer: You described how DBS has made her more positive as she is able to do more, can you now describe what it has been like for you?*

*H1Y5: Well again I know I keep talking about her I suppose but I was pleased for her I was pleased that she felt this way and I still walk too fast, so I haven't really got much out of it but I was very pleased for her that she was getting this treatment.”*

## 4. Discussion

### 4.1. The Issues with Self-Disclosure

Conducting this study was not without its challenges and those challenges have raised some questions which are worthy of discussion. The first challenge of note is the issue that arose during data collection and analysis. Half of the 16 participants did not share enough reflections regarding their own lived experience for an IPA analysis. They disregarded, deflected, or chose not to answer many questions about their experience, and chose to speak predominantly about their ill partners. This could be indicative of something personal that was playing out for these carers. Carers can feel invisible [16], and these carers may have been uncomfortable with the level of visibility an interview afforded them, but it would be wrong to make any assumptions as to why they shared so few reflections, with such a small amount of data.

The method of data collection may have also impacted on the participants' ability to share. Other studies have used multiple interviews as a means of building rapport and engendering psychological safety [30, 31] to allow for deeper levels of self-disclosure. By the time it became clear that this was an issue within the data, the UK had been locked down due to the COVID-19 pandemic. The decision was made to continue analysing the data as collected, as interviewing caregivers virtually via Zoom or Microsoft Teams, with their spouses under the same roof, could potentially create new barriers to self-disclosure. Considering the current events, it would have also been difficult to compare the lived experience of caregivers in lockdown, to those who had been interviewed 4–6 months prior. If this study were to be replicated, consideration should be given to using a series of multiple interviews.

For as much as one's identity is created through one's own narrative, it also has a social context [32]. Individuals make sense of themselves through their interactions with others. By placing all attention on their partners, the thoughts and feelings about their own experiences were lost to the interviewer. This behaviour could suggest elements of “role engulfment and loss of self” which occurs “when the role of caregiver and responsibilities of caring begin to consume a person, leaving little time for other activities and behaviours that may have defined the person previously” [33]. This was prevalent in all the caregivers in group 2. In line with the caregiver identity theory [15], the introduction of an intervention such as DBS should have helped these caregivers to shift their attention away from their partners and more on to themselves, and yet this shift was not evident at interview, a phenomenon which is worthy of note and further exploration. This shift was however, present in the remaining 8 caregivers in group 1.

### 4.2. Shifts in Caregiver Identity: DBS Creates Possibilities

In line with the proposed stages operational post DBS [8], all of the caregivers in group 1 were open to the shifts in their roles thanks to the changes in their spouses' symptoms and behaviours. We see from the first theme “DBS allows carers to question and shift the caregiver role” an evolution occurring between the two time points. The subtheme for the 4 participants left in the 12–18 months postsurgery group is, *Am I more than just a caregiver?* It has been noted that in the first year post DBS surgery, a positive result can give patients and spouses a sense of liberation and a less favourable result can bring on the need to reconcile with disappointment [31]; Haahr et al. [21]. These emotions were in part reflected in the testimonies of the participants.

W4Y1's anger when she stated, “*it's not just him that's got Parkinson's and I could, I could have not stopped working, I could have carried on working,”* shows her awareness of the choice she made to step into her caring role as she describes her disappointment that DBS has not given her any respite. It has been noted before that women struggle more with lack of freedom and excessive demands being placed on them by their partner's disease progression [34]. Whereas, all the caregivers in this group seemed to show some level of role engulfment, W4Y1 is not so engulfed that she is unaware of the lack of freedom her caregiving role affords her. We sense that this may be why she is associating with the role of the patient, as it offers her more than her caregiver role.

H1Y1 seemed much more comfortable and welcoming of the engulfment of his caregiver role. This could fit with Hughes' theory of master identity [35]. The master identity is formed when other identities are lost and the prevailing identity, in this case the role of caregiver, overshadows all others. H1Y1 explained that he can only see himself as a caregiver. Once faced with the potential loss of that identity we can postulate that his fear was triggered by the “burden of normality” [6] that has been described by patients post-DBS. Adjusting back to a more “normal” way of living can be daunting, particularly when it affects one's identity. H1Y1 is very comfortable in his role and feels united with his wife in their management of PD, DBS could shift that dynamic.

### 4.3. Shifts in Caregiver Identity: The Separation of Self

The second theme “Parkinson's unites and DBS divides” explores this shift further. H3Y1 refers to his “combined self,” a concept which has been seen in other studies (Haahr et al. [21]) and was alluded to by all of the other caregivers in the earlier group. In the first year post DBS, the master identity of caregiver, if indeed one has arisen, has the potential to be challenged if the person with PD receives benefit from DBS surgery. The 4 of the 8 caregivers interviewed 4-5 years post DBS did not allude to this sense of unity. They felt more comfortable asserting new identity roles. W1Y5 referred to this as “reclaiming her life” which could be translated to, she reclaimed her other identities.

H4Y5 displays how his wife's reengaging in activities in the home has facilitated his ability to have more control over his own life. He is at ease with reclaiming his life. It has been reported that some people with PD struggle to involve themselves in activities of daily living post-DBS surgery which can lead to marital dissatisfaction [36]. H4Y5 reported feeling closer to his wife because she is engaging more in her role of partner, DBS has helped this couple to divide themselves off from the roles of caregiver and patient.

Feeling a sense of control while living with illness has been shown to improve life satisfaction and depression in caregivers [37]. People with an internal locus of control are motivated to engage in efforts which allow them that sense of control. They are also more likely to profit from psychological interventions [38]. This can lead us to the question of whether they could also benefit from surgical interventions such as DBS. These 8 participants, whose interviews were analysed, suggest that DBS has restored a sense of control back to them, improving their sense of wellbeing and their sense of self.

### 4.4. Shifts in Caregiver Identity: Remembering One's Self

The third theme, “seeing myself and my needs, DBS enhances visibility” encompasses the two subthemes, “*I'm still invisible, DBS hasn't helped enough” and* “*Acceptance means it's easier to live for me*”. Unlike previous studies that have shown a direct correlation between the spouse's treatment success and the caregiver's change in life experience (Haahr et al. [21]), the results of this study highlight the harsh reality that sense of self is not only dependent on external factors such as the caree's state of health. The results of DBS surgery vary from individual to individual, a reality that was very much captured by the experiences of all 16 participants. For the four caregivers who contributed to the subtheme of invisibility, elements of role engulfment still seemed visible, hence H3Y1's comment, “*the best bit (about DBS) really is, is seeing her life get better.”* That shift from phase four of caregiver identity, where the caregiver's lives are heavily dominated by their caregiving role, to phase five, where the individual reverts back to an earlier phase of identity [15], is starting to occur but it seems to be still in process.

The caregivers in the second group all seemed more in line with phases 2 or 3 of the caregiver identity theory. They were still involved in some of their partner's care but had a greater sense of self even though their spouses were starting to experience more PD symptoms. This may be described as redefining PD, something which occurs after the first year after surgery, once when both spouses find new ways to interact with PD due to the biographical shift caused by DBS [31]. These caregivers displayed good coping mechanisms such as acceptance of their partners' state of health and seeking out social support. Improving coping strategies through a short course of cognitive behavioural therapy one year post surgery has been shown to benefit caregivers post DBS for PD [39]. The caregivers who were operated 4-5 years prior have a better sense of self than those interviewed at the earlier time point.

### 4.5. Creating Future Resources

Many research teams have mentioned the need for pre and postoperative support for couples who embark on the DBS journey [36, 40, 41]. Attention is often given to the impact of expectations on satisfaction and burden [42, 43] with suggestions for interventions prior to DBS aimed at managing expectations and nurturingcoping strategies [44]. Once more, this shows a bias towards the management of physical symptoms post DBS.

Managing PD is a complex process. The progression of the disease can cause one's sense of self and identity to shift during its life course (Haahr et al. [45]). DBS surgery is one more shift which must prompt more changes the within the self. Using the triadic model of multiple conversations with a nurse is worthy of consideration. Individualised meetings among nurse, patient, and spouse have been shown to shift the focusing on the physical adjustment process post DBS to the emotional adjustment process (Haahr et al. [20]). Repeated meetings have shown to create a safe space, where both members of the couple are invited to share their perceptions around everyday life, coping strategies, and expectations, allowing not only for them to be understood and guided by the DBS nurse but also giving the couple time to appreciate and have deeper understanding of each other. When we consider the testimonies of W4Y1 and H3Y1, we sense an imbalance in these caregiver's relationships with their spouses, as so much attention is focused on their spouse. This manner of intervention can bring balance to how the couple manage the adjustment process post surgery by giving each of them equal importance, time, and space to share. Individual therapy may also be considered as it can allow caregivers time to reflect and express their feelings, separate from their caree. In this case, it could help these spouses to process and separate out their own life journey from their partners. The results of this study suggest further explorations of the self-identity of the patient and the caregiver following future interventions, may be of value.

Health professionals could also consider approaches from positive psychology for innovative ways of helping caregivers to redefine their sense of self. Character strength interventions, whereby participants are asked to engage in those aspects of themselves, such as love of learning or creativity, can enhance pleasure and meaning independent of the caring role [46]. Facilitating access to psychological therapy, discussing the mobilization of social support networks and preoperative discussions around “readjustment” should all be considered [4].

## 5. Conclusions

DBS has the potential to restore a sense of self and agency, as displayed by half of the caregivers interviewed. However, post DBS, itcan take time for spouses to make the shift from identifying as caregiver back to seeing themselves as partner.

After years of various patterns of behaviour becoming the norm within the relationship between people with PD and their spouses, DBS may offer caregivers the opportunity to create new behavioural patterns and those behavioural shifts can help give rise to new ways of identifying. As described previously, the handing of control and responsibility back to their spouses, was a process which many caregivers faced, which can cause distress for both partners at the beginning of their DBS journey. The more we understand these dynamics, the more clinical teams can support couples in relation to the emotional and psychological shifts that can occur post DBS, alongside the physical changes in the PD patient.

The differences in identity between the two subgroups of caregivers in group 1, supports the notion that identity is something that evolves over time. Healthcare providers should consider having conversations around identity, at various moments in time, to help support the shifts that occur for both the person with PD and the caregiver. Whether it is at the beginning of the PD journey, or at seminal moments such as pre- or post-DBS surgery, awareness is key. Making both parties aware of the potential effects PD can have on roles and responsibilities within the home and within one's partnership can help couples to behave more mindfully when possible, allowing for more control over any identity shifts.

This was a small study, and the ability to recruit more caregivers was cut short by the COVID-19 pandemic. Nevertheless, the results suggest a need for further exploration of the impact of DBS on caregiver identity and the effects of time on their psychosocial adjustment post surgery. That half the spouses found it hard to discuss their own reality is concerning and worthy of note. It suggests that some caregiving partners need substantial encouragement and support in understanding their own lived experiences prior to and post DBS. If caregiving spouses understand their identity and recognise their needs, outside of their caregiving roles, this may help facilitate psychosocial adjustment post surgery, not only for themselves but also for their spouse who has PD.

### 5.1. A Reflexive Statement from the First Author

I have been intimately connected to PD for over 20 years, and still I am learning about the effects of this illness. I am a therapist who has worked with people with PD and their caregivers for over 10 years. I have also been a caregiver, one who cared for a husband with PD for over 20 years and supported him through the DBS experience. One of the challenges I feared facing, in this research project, is keeping the balance between using my knowledge to inform my work, without projecting my life experience onto it.

In IPA, we speak about convergence and divergence. We look for the similarities that unite personal experience, while also looking for the differences that keep those experiences unique. This project had me reflecting consistently on those convergences and divergences. So, many of my own life experiences were mirrored in the testimonies shared, yet the way in which each individual described those life experiences and the impact they had; they were so different to my own journey.

When you live PD, and yes I mean live PD, one does not live with PD as if it is an addition to one's life; one eats, sleeps, and breathes PD. When you live PD, you start to recognise that no matter how much you have in common with others who live PD, your experience will always be unique and that can be isolating. So, we turn to others and look for those convergences, those strands of commonality that help us to feel like we are not alone in our suffering. We take comfort that someone recognises some of our challenges, and we give gratitude that there are resources that address those issues that so many of us share.

Interviewing this amazing cohort of caregivers was a privilege and a humbling experience, and I am grateful to all the participants who gave up their time to share their experiences with me so that I can help others understand them enough to help them through their challenges. I am also incredibly grateful to the other authors and my colleagues, who supported me through this study as they helped me to disentangle my own emotions and focus on what's important.

## Figures and Tables

**Table 1:** Breakdown of how superordinate theme 1 from husband 3 year 1's interview evolved.

Themes IPA interview table husband 3 year 1	Keywords taken from transcript
Superordinate theme 1: if she is well, I am well	
PET1: living a combined life	
Prior to and post DBS, he feels the effects of PD so much he feels as if he has it too	I do not think of myself as (a carer) It is just our life… We actually say, you know, we have got Parkinson's, if you like so it is more inclusive
His life is very entwined with hers, DBS makes their conjoined lives better now but he cannot stop their combined life from declining	It is like a combined life, is how I see it really We thought well this is not working very well so we will do it ourselves
PET 2: the caring carer irrespective of DBS	
He likes how well he cares for her and is proud that he maintained his relationship as a husband rather than carer prior to DBS	I have to help with all that… I do not always wait to be asked either, it is quite a sort of delicate…
He desperately wants to ease as much of her suffering as possible and fears that he is not able to post DBS	I worry sometimes, I am not being thoughtful enough, have missed something… anxious I: is that even now after DBS? H3Y1: generally, yeah

**Table 2:** Number of PES's per participant.

Group 1 participant	Number of PES's	Group 2 participant	Number of PES's
W2Y1	51	W1Y1	23
W4Y1	66	W3Y1	26
H1Y1	76	H2Y1	29
H3Y1	53	H4Y1	13
W1Y5	54	W2Y5	29
W4Y5	52	W3Y5	25
H3Y5	66	H1Y5	24
H4Y5	50	H2Y5	18

**Table 3:** Themes and subthemes by group.

Theme 1: identity	DBS allows carers to question and shift the caregiver role
Group A (12–18 months)	Am I more than just a caregiver?
Group B (years 5)	The carer identity has shifted
Theme 2: independence	Parkinson's unite and DBS helps divide in time
Group A	The combined self
Group B	DBS brings balance in relationships through acceptance
Theme 3: personal needs	Seeing myself and my needs, DBS enhances visibility
Group A	I am still invisible, DBS has not helped enough
Group B	Acceptance means it is easier to live for me

**Table 4:** Strategies used by participants in group 2 when answering questions.

Strategy 1	A bias when answering the opening question
Strategy 2	Continuation of bias when answering subsequent questions
Strategy 3	Awareness of bias when answering questions

**Table 5:** Strategies used by participants answering first question.

	Ability to reflect on first question	Ability to reflect and answer once question was repeated	Reflected once question was repeated but reverted to avoidance again	Deflected	Avoided answering once question was repeated
Number of participants group 11 year post DBS	4	N/A	N/A	N/A	N/A
Number of participants group 11 year post DBS	4	N/A	N/A	N/A	N/A
Number of participants group 21 year post DBS	0	0	2	0	2
Number of participants group 25 years post DBS	0	1	0	1	2

## Data Availability

Access to patient interview transcripts is restricted due to patient confidentiality.
